# SenMayo Based Aging Signature (Sen-Age) in cancer survivors: The Health and Retirement Study (HRS)

**DOI:** 10.1093/geroni/igaf122.2967

**Published:** 2025-12-31

**Authors:** Gokul Seshadri, Shuo Wang, Weihua Guan, Bharat Thyagarajan, Anna Prizment

**Affiliations:** University of Minnesota, Minneapolis, Minnesota, United States; University of Minnesota, Minneapolis, Minnesota, United States; University of Minnesota, Minneapolis, Minnesota, United States; University of Minnesota, Minneapolis, Minnesota, United States; University of Minnesota, Minneapolis, Minnesota, United States

## Abstract

The SenMayo gene set identifies senescent cells, one of the hallmarks of aging, especially among those with cancer. In the HRS cohort, we constructed a SenMayo based aging signature (Sen-Age) among those without cancer (controls) and examined its association with 4-year mortality in cancer survivors. In the 2016 wave, HRS measured 125 SenMayo genes in blood samples. We applied Lasso regression to participants without cancer in 2016 (N = 3076) and trained SenMayo genes against chronological age; this resulted in a subset of 97 genes with which we created Sen-Age. This Sen-Age signature demonstrated a correlation of 0.46 with chronological age in 609 cancer survivors. We then examined the associations of Sen-Age with 4-year mortality (N = 112) in cancer survivors using Cox Proportional Hazards regression model after adjusting for age, sex, and race. Sen-Age was associated with increased risk of mortality in cancer survivors (HR: 1.47 [1.17–1.85] per 1 year, p = 0.0008). This association remained robust (HR: 1.31 [1.04–1.66], p = 0.02) upon additional adjustment for education level, smoking status, BMI, inflammation and comorbidities. Moreover, cancer survivors have higher Sen-Age score (adj mean: -0.44 ) than non-cancer controls (adj mean: -0.83). Overall, our findings highlight the potential of the Sen-Age signature as a biomarker of aging and mortality in cancer survivors.

